# Predicting Effects of Tropomyosin Mutations on Cardiac Muscle Contraction through Myofilament Modeling

**DOI:** 10.3389/fphys.2016.00473

**Published:** 2016-10-26

**Authors:** Lorenzo R. Sewanan, Jeffrey R. Moore, William Lehman, Stuart G. Campbell

**Affiliations:** ^1^Department of Biomedical Engineering, Yale UniversityNew Haven, CT, USA; ^2^Yale School of Medicine, Yale UniversityNew Haven, CT, USA; ^3^Department of Biological Sciences, University of Massachusetts LowellLowell, MA, USA; ^4^Department of Physiology and Biophysics, Boston University School of MedicineBoston, MA, USA; ^5^Department of Cellular and Molecular Physiology, Yale School of MedicineNew Haven, CT, USA

**Keywords:** tropomyosin, tropomyosin-actin interactions, tropomyosin stiffness, cooperativity, hypertrophic cardiomyopathy, computational modeling, diastolic dysfunction

## Abstract

Point mutations to the human gene TPM1 have been implicated in the development of both hypertrophic and dilated cardiomyopathies. Such observations have led to studies investigating the link between single residue changes and the biophysical behavior of the tropomyosin molecule. However, the degree to which these molecular perturbations explain the performance of intact sarcomeres containing mutant tropomyosin remains uncertain. Here, we present a modeling approach that integrates various aspects of tropomyosin's molecular properties into a cohesive paradigm representing their impact on muscle function. In particular, we considered the effects of tropomyosin mutations on (1) persistence length, (2) equilibrium between thin filament blocked and closed regulatory states, and (3) the crossbridge duty cycle. After demonstrating the ability of the new model to capture Ca-dependent myofilament responses during both dynamic and steady-state activation, we used it to capture the effects of hypertrophic cardiomyopathy (HCM) related E180G and D175N mutations on skinned myofiber mechanics. Our analysis indicates that the fiber-level effects of the two mutations can be accurately described by a combination of changes to the three tropomyosin properties represented in the model. Subsequently, we used the model to predict mutation effects on muscle twitch. Both mutations led to increased twitch contractility as a consequence of diminished cooperative inhibition between thin filament regulatory units. Overall, simulations suggest that a common twitch phenotype for HCM-linked tropomyosin mutations includes both increased contractility and elevated diastolic tension.

## Introduction

Point mutations in alpha tropomyosin (TPM1) are associated with inherited cardiomyopathies, most notably hypertrophic cardiomyopathy (HCM) and dilated cardiomyopathy (DCM) (Redwood and Robinson, 2013). While there is no consistent pattern predicting whether amino acid substitutions will lead to HCM versus DCM, mutations are known to alter the interactions within the tropomyosin coiled-coil itself and between tropomyosin and its binding partner actin (Bai et al., 2013; Redwood and Robinson, 2013). Studies on the classic HCM-related tropomyosin mutants E180G and D175N (Thierfelder et al., 1994) using electron microscopy, atomic force microscopy, and molecular dynamics on isolated tropomyosin have shown increased local and global flexibility (Li et al., 2010, 2012; Loong et al., 2012b). Changes in tropomyosin flexibility are widely believed to affect thin filament cooperativity (Loong et al., 2012a; Moore et al., 2016). However, recent work has shown that point mutations in tropomyosin can also affect the energy landscape of tropomyosin interactions with actin, providing another potential route for mutations to affect thin filament calcium regulation (Orzechowski et al., 2014a,b). Altered actin binding by mutant tropomyosin is also supported by numerous experimental and computational studies (Boussouf et al., 2007; Janco et al., 2012; Zheng et al., 2016). The surface interactions of tropomyosin and actin may ultimately affect myosin crossbridge formation, as suggested by length perturbation analysis studies (Bai et al., 2011). Based on these observations, we posit that emergent physiological effects of tropomyosin mutations can be described by considering the effects of residue changes on both the chain-like properties of the tropomyosin molecule and interaction of tropomyosin with the actin surface as it fluctuates between regulatory states.

In order to construct a paradigm for exploring the effects of tropomyosin properties on muscle activation, we employed a computational approach. Beginning with a previous model of thin filament calcium activation (Aboelkassem et al., 2015), we reconsidered the formulation of tropomyosin neighbor interactions, bringing it into closer agreement with structural measurements of tropomyosin movement on actin. The completed model includes parameters that unambiguously relate to three potential mutation-dependent molecular properties of tropomyosin, namely tropomyosin chain stiffness, blocked-closed equilibrium, and the crossbridge duty cycle. Making use of published data, we found that the model was capable of capturing steady-state behavior of cardiac muscle preparations containing wild-type and mutant tropomyosins. Furthermore, the simulations suggest that HCM-related tropomyosin mutations produce hypercontractile twitch phenotypes with diastolic dysfunction.

## Methods

### Model formulation

**Figure 1:**
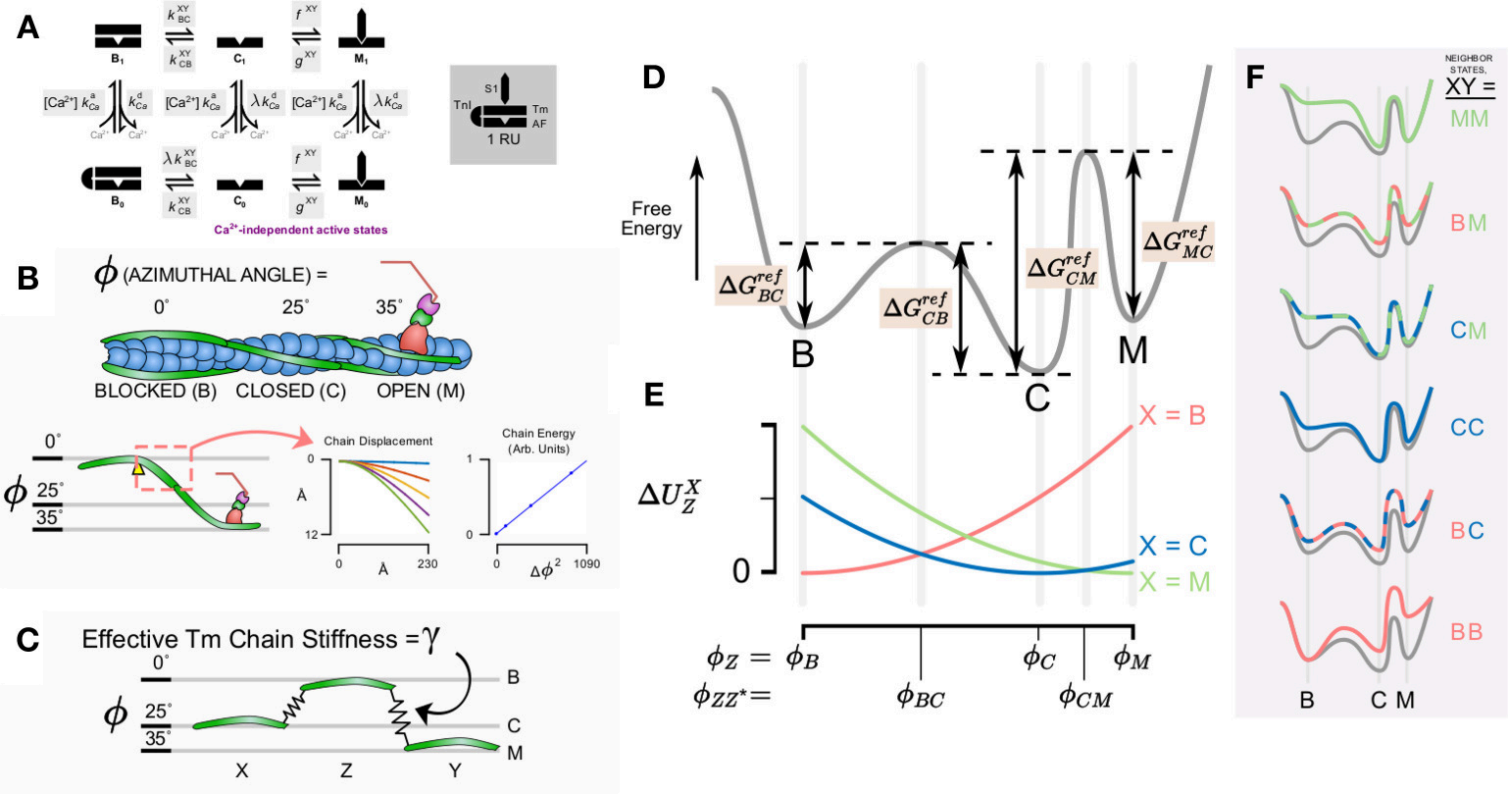
**Schematic diagram of model components, states, and conceptual underpinning of the cooperativity scheme. (A)** State diagram for the assumed six states in the thin filament model with kinetic rates governing each state transition (shaded). **(B)** The model takes into account the azimuthal angle displacement required for the state transition from blocked to closed to open for a given regulatory unit. Quasi-explicit representation of Tm bending shows that stored energy in the molecule is proportional to the square of the azimuthal angle change. **(C)** This suggests that the system can be represented simply as a given regulatory unit Z connected to its neighbors X and Y by linear springs. **(D)** The conceptual reaction coordinate diagram for a given regulatory unit detached from its neighbors considers the free energy barrier from the blocked state to closed state to open state. **(E)** Magnitude of strain energy contribution for a given neighbor X depends on the intermediate angles between a transition for the state of a given regulatory unit and the state of the neighbor. **(F)** The overall energy barriers for the transitions of a given regulatory unit depends on the contribution of the strain energy with respect to the states of its neighbors X and Y.

Consider an actin regulatory unit (RU) having a current state *Z* ∈ {B, C, M} after the three state model established by McKillop and Geeves (1993) (Figures 1A,B). In order to transition to a different state, Z^*^, the RU must overcome an activation energy which we denote $\Delta G_{ZZ^{*}}^{ref}$.

Because direct transitions between B and M are prohibited (Figure 1A), this yields a total of four reference activation energies, $\Delta G_{BC}^{ref}$, $\Delta G_{\text{CB}}^{ref}$, $\Delta G_{\text{CM}}^{ref}$, and $\Delta G_{\text{MC}}^{ref}$ (Figure 1D). These energies correspond to theoretical reference conditions in which the RU is not connected to its nearest neighbors. From these, it is possible to construct a baseline free energy landscape for RU transitions (depicted conceptually as the gray line in Figure 1D).

Having established a reference free energy landscape, the effects of nearest neighbors can be evaluated. Consider a thin filament formed of discrete RUs connected in series (Campbell et al., 2010). Each RU has structural links with its neighbors through tropomyosin-tropomyosin overlap along the thin filament (McLachlan and Stewart, 1975). We include the effect of Tm-Tm overlap by determining the potential energy stored in the entire Tm chain itself when adjacent RUs do not occupy the same state (Figures 1B,E). The potential energy is assumed to arise from distortion of the Tm chain according to the difference in azimuthal angle between adjacent Tm. In preliminary calculations, we determined the energy stored in an elastically jointed chain cantilevered at one end and loaded (orthogonal to the un-deformed chain) at the other (Figure 1B). We found that the total chain energy was proportional to the square of the azimuthal angle change subtended by the bending chain. In terms of stored energy, this result is equivalent to a virtual spring element connecting RUs azimuthally. Accordingly, rather than represent a continuous chain in the model, we adopted a mechanical analog in which each Tm was considered attached to its neighbors via a linear elastic element (Figure 1C). When adjacent RUs occupy the same state, the distortion in their linking spring and its developed elastic force is zero. When they occupy different states, the force is proportional to the azimuthal angle (ϕ) that separates them. The constant of proportionality is the effective Tm chain stiffness γ. Hence, the potential energy contributed to an RU by its left neighbor (having state *X* ∈ {B,C,M}) is:

(1) $$\Delta U_{Z}^{X}=\frac{1}{2}\gamma {\left(\phi_{Z}\;-\;\phi_{X}\right)}^{2}$$

The same equation applies analogously to the right neighbor, having state *Y* ∈ {B,C,M}:

(2) $$\Delta U_{Z}^{Y}=\frac{1}{2}\gamma {\left(\phi_{Z}\;-\;\phi_{Y}\right)}^{2}$$

The azimuthal angles of tropomyosin for each state are taken from structural data (Vibert et al., 1997; Poole et al., 2006), namely $\phi_{B}=0^{{}^{\circ}}$, $\phi_{C}=25^{{}^{\circ}}$, and $\phi_{M}=35^{{}^{\circ}}$. We further assume that the peak free energy barrier between states coincides with an azimuthal angle precisely halfway between the angles associated with each state, that is:

$$\begin{array}{l} \phi_{BC}=\phi_{CB}=\frac{1}{2}\left(\phi_{C}\;-\;\phi_{B}\right)+\;\phi_{B} \end{array}$$

and

$$\begin{array}{l} \phi_{CM}=\phi_{MC}=\frac{1}{2}\left(\phi_{M}\;-\;\phi_{C}\right)+\;\phi_{C} \end{array}$$

The potential energy due to Tm chain distortion at these intermediate angles, accounting for the left-neighbor state *X* is:

(3) $$\Delta U_{ZZ*}^{X}=\frac{1}{2}\gamma {\left(\phi_{ZZ*}\;-\;\phi_{X}\right)}^{2},$$

where *Z* is the current state of the RU, and Z^*^ is the state into which the RU is transitioning. Similarly, the potential energy contribution of the right neighbor (having state *Y*) is

(4) $$\Delta U_{ZZ*}^{Y}=\frac{1}{2}\gamma {\left(\phi_{ZZ*}\;-\;\phi_{Y}\right)}^{2}$$

The final activation energy for any given transition ZZ^*^ with neighboring RU states *X* and *Y* is obtained by altering the reference activation energy as follows:

(5) $$\begin{array}{l} \Delta G_{ZZ^{{}^{*}}}^{XY}=\Delta G_{ZZ^{{}^{*}}}^{ref}\;-\;\Delta U_{Z}^{X}\;+\;\Delta U_{ZZ^{{}^{*}}}^{X}\;-\;\Delta U_{Z}^{Y}\;+\;\Delta U_{ZZ^{{}^{*}}}^{Y} \end{array}$$

In other words, distortion-based increases in potential energy raise energy wells, hence the two negative terms in the above equation that diminish the reference activation energy. On the other hand, distortion increases the height of the peaks in between states, adding to the activation energy. This explains the positive potential energy terms.

Once activation energies are defined, neighbor-dependent kinetic rates can be obtained via the Eyring equation:

(6) $$k_{ZZ^{{}^{*}}}^{XY}=\frac{k_{b}T}{h}e^{\;-\Delta {\text{G}}_{{\text{ZZ}}^{{}^{*}}}^{\text{XY}}/RT}$$

For convenience, we define a reference kinetic rate based on the reference energy change $\Delta G_{ZZ^{*}}^{ref}$:

$$k_{ZZ^{\text{*}}}^{ref}=\frac{k_{b}T}{h}e^{\;-\Delta {\text{G}}_{{\text{ZZ}}^{\text{*}}}^{\text{ref}}/RT}$$

Using Equation (5), $k_{ZZ^{*}}^{ref}$ can be substituted into Equation (6):

(7) $$\begin{array}{l} k_{ZZ^{*}}^{XY}=k_{ZZ^{*}}^{ref}e^{\left(-\Delta U_{Z}^{X}\;+\;\Delta U_{ZZ^{*}}^{X}-\Delta U_{Z}^{Y}\;+\;\Delta U_{ZZ^{*}}^{Y}\right)} \end{array}$$

Equation (7) casts tropomyosin transition rates as a reference kinetic rate scaled by a term that reflects that status of nearest neighbors as well as the apparent tropomyosin chain stiffness (Equations 1–4). In order to conform to nomenclature of previous models, C → M and M → C transition rates are referred to as *f* and, *g* as these transitions are driven by crossbridge binding and unbinding, respectively:

$$\begin{array}{l} \begin{array}{c} {f^{XY}=k}_{CM}^{XY}\\ {g^{XY}=k}_{MC}^{XY} \end{array} \end{array}$$

In addition to tracking the tropomyosin state of each RU, we also represent Ca^2+^ binding to TnC. As introduced in Aboelkassem et al. (2015) we permit all three tropomyosin RU states to be either Ca^2+^ bound or Ca^2+^ free (denoted by subscripts 1 or 0, respectively). This produces a six-state RU model (Figure 1A). In order to reflect the fact that transition away from the B state seldom occurs without Ca^2+^ first binding to TnC, the kinetic rate $k_{BC}^{XY}$ is scaled by the factor λ for the transition B_0_ → C_0_. The Ca^2+^ dissociation rate $k_{Ca}^{d}$ is also scaled by λ for C_1_ → C_0_ and M_1_ → M_0_ transitions in order to satisfy microscopic reversibility (Aboelkassem et al., 2015). Ca^2+^ association with the RU is governed by the second order rate constant *k*_*Ca*+_.

### Model implementation

A total of 24 RUs, each obeying the six-state scheme described above, were connected in series to form a virtual thin filament. Dummy RUs, one on each end of the filament, were added and permanently fixed in the B_0_ position to define boundary conditions. Simulations were performed on the system using a Markov chain Monte Carlo algorithm described previously (Aboelkassem et al., 2015). Force produced by the model was also computed as before by averaging the output of many repeated simulations. The time step size for simulations was automatically determined for each parameter set such that the maximum cumulative probability of transition for any combination of nearest neighbor states never exceeded 0.7. To guarantee the accuracy of this heuristic threshold, simulations were tested for convergence at other time step values. The 0.7 transition threshold proved more than sufficient to guarantee temporal convergence in each case.

**Figure 2:**
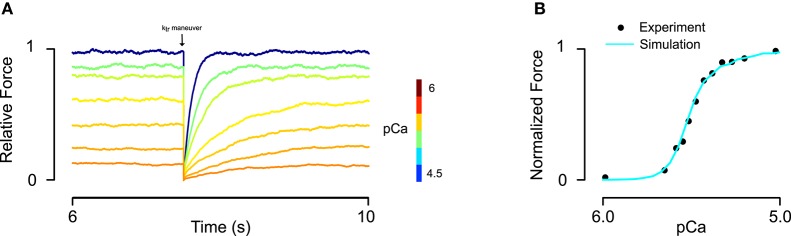
**Fitting of skinned rat trabeculae steady state force-pCa data. (A)** The model showed realistic calcium dependence of steady-state force and the rate of force development (*k*_tr_) after a slack-restretch maneuver. **(B)** The model was able to fit steady-state force-pCa data and recapitulate the asymmetric Hill coefficient observed in experiments (data from Dobesh et al., 2002).

Simulation protocols included both activations at a single, constant Ca^2+^ concentration and under time-varying intracellular Ca^2+^ concentration (for twitch). Data for fitting steady-state and twitch activation (Janssen and de Tombe, 1997; Dobesh et al., 2002; Bai et al., 2011) were digitized using a custom script that loaded a bitmap of each figure into MATLAB and located the pixel corresponding to the centroid of each data markers. Digitized data points were then overlaid on the original images to check for fidelity. RUs were always set to an initial state of B_0_ at the beginning of each simulation. In order to obtain steady-state force and rates of tension recovery (*k*_tr_), the model was run for a 7500 ms interval (achieving steady force) and then all RUs in M states were instantaneously transitioned into C states. The rate at which force recovered to the steady-state value was used to determine *k*_tr_ (Figure 2A). The model was implemented in CUDA C++ and executed on an Nvidia Tesla K40 graphic processing card. Custom MATLAB scripts were created to manage simulation setup and data flow.

## Results

Implementation of the new formulation (Figure 1) of tropomyosin nearest neighbor interaction constrained by biophysical measurements on tropomyosin azimuthal change (Poole et al., 2006) decreases the number of free parameters of the model by four. This constitutes a drastic reduction in potentially adjustable parameters and in model dimensionality compared to the previous formulation (Aboelkassem et al., 2015). To determine whether the model could still lead to behavior consistent with realistic calcium activation of muscle, we used constrained particle swarm optimization to find a set of parameters (Table 1, Set 1) that accurately matched calcium-dependent steady-state force production reported in skinned rat trabeculae (Figure 2; Dobesh et al., 2002). The dataset of Dobesh et al. (obtained at a sarcomere length of 2.25 μm) was selected because of the meticulous attention paid to sarcomere length stability in that study. The simulation reproduced not only the steady state force-pCa relationship as reported (Figure 2B) with roughly the same Hill coefficient and pCa_50_ over the experimental pCa range (Table 2) but also showed a strong dependence of the rate of force redevelopment on pCa (Figure 2A), consistent with experimental observation (Fitzsimons et al., 2001). The simulation furthermore manifested asymmetry in the lower half of the force-pCa relationship compared to the upper half such that a higher Hill coefficient can be calculated at the lower half than the upper half (Figure 2B). While an asymmetric cooperativity leads to deviation of the simulation curve from an idealized Hill curve with the same apparent cooperativity, real muscle including the skinned trabeculae modeled here defies uniform cooperativity in its force-pCa relationship (Dobesh et al., 2002).

**Table 1:** **Parameters used for fitting data and running simulations**.

**Parameter**	**Set 1 Skinned filament (Dobesh et al., 2002)**	**Set 2 Reconstituted myocardium (Bai et al., 2011)**	**Set 2 w E180G changes**	**Set 2 w D175N changes**	**Set 3 Intact rat trabeculae (Janssen and de Tombe, 1997)**	**Set 4 Intact rat trabeculae w E180G changes**	**Set 5 Intact Rat Trabeculae w D175N changes**
*k_Ca_*−(*ms*^−1^)	0.58	0.471			6.0		
*k_Ca_*+(*uM*^−1^*ms*^−1^)	0.125	0.1003			6.0		
*K_BC_*	0.898				7.0		
*$k_{BC}^{ref}$*(*ms*^−1^)	1.83	1.9524	2.1476	1.6595	1.2963	1.4259	1.212
γ(*mol*^−1^*kJ*)	68.00	37.64	28.20	22.60	68.00	51.00	40.80
*f^ref^*(*ms*^−1^)	0.0058				0.132		
δ	0.4754	0.4951	0.5694	0.4951	0.715	0.822	0.715
λ	0	0.20			0.06		

**Table 2:** **Tropomyosin wild-type fit simulation and data properties**.

**Parameter**	**Experimental average data (Bai et al., 2011)**	**Simulated curve**	**Experimental average data (Dobesh et al., 2002)**	**Simulated curve**
*nH*	1.78	1.91	6.9	6.7
*pCa*50	6.0	6.0	5.5	5.5

**Figure 3:**
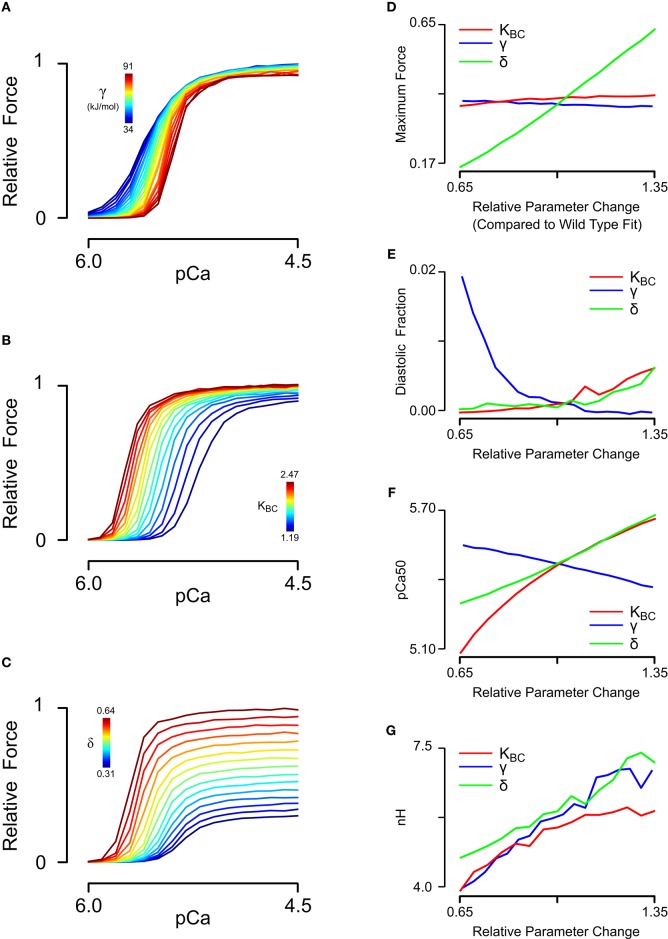
**Sensitivity analysis of steady-state force-pCa simulations. (A)** Relative steady state force-pCa curves as a function of the tropomyosin stiffness varied from 50 to 135% of the original fit stiffness of 68e3. **(B)** Relative steady state force-pCa curves as a function of the BC equilibrium constant varied from 65 to 135% of the original fit BC equilibrium constant of 1.83. **(C)** Relative steady state force-pCa curves as a function of the duty cycle varied from 65 to 135% of the original fit duty cycle of 0.47. **(D)** The maximum steady state force production depended primarily on the myosin duty cycle while BC equilibrium and stiffness have minor effect. **(E)** The fraction of the maximum steady state force present at low calcium (pCa 6.0) was strongly shaped by the stiffness with a minor dependence on the BC equilibrium and duty cycle. **(F)** Whereas, increased BC equilibrium and duty cycle increase the pCa_50_, stiffness served to decrease the pCa_50_. **(G)** Cooperativity could be strongly tuned by variation in tropomyosins stiffness and myosin duty cycle whereas BC equilibrium could decrease cooperativity but could not increase it much above its steady state value.

Having obtained a parameter set for skinned cardiac muscle, we perturbed each of the model parameters that could potentially be affected by tropomyosin mutations (Li et al., 2012; Bai et al., 2013; Orzechowski et al., 2014a) in order to understand their separate effects on the steady state force-pCa relationship (Figure 3). The same relative changes in tropomyosin stiffness γ, BC equilibrium constant *K*_*BC*_, and myosin duty cycle δ were used to generate force-pCa curves (Figures 3A–C) with their properties quantified as maximum force, fraction of maximum force present at diastolic (low) calcium, calcium sensitivity, and Hill coefficient. The three troponin-related parameters (λ, *k*_*Ca*+_, *k*_*Ca*−_) were not perturbed in this analysis. The forward rates of BC transition and of myosin binding were also excluded because the absolute rates have little effect on the steady state force-pCa curves (data not shown). The maximum steady state force production was strongly dependent on the myosin duty cycle whereas tropomyosin stiffness and BC equilibrium had only small effects on the maximum force (Figure 3D). Examining the effect closely of stiffness and BC equilibrium revealed that they had primarily fine tuning but opposing effects on maximum force production, with higher stiffness slightly decreasing force production and higher BC equilibrium slightly increasing force production. The force present at low calcium (Figure 3E) was most sensitive to tropomyosin stiffness. Decreasing the stiffness parameter causes a drastic, non-linear increase in force at low calcium. Proportional increases in BC equilibrium and duty cycle increased force at low calcium, but these changes were modest by comparison (Figure 3E). While increases in BC equilibrium constant and duty cycle tended to increase the calcium sensitivity of the thin filament in these simulations (Figure 3F), increased stiffness had the opposite effect. BC equilibrium had a larger effect on calcium sensitivity than duty cycle. Cooperativity (as assessed by the Hill coefficient) was affected by all three parameters, but more particularly myosin duty cycle and tropomyosin stiffness (Figure 3G). Specifically, a higher BC equilibrium resulted in just a modest increase in cooperativity but ultimately reached a plateau. Of the three parameters, tropomyosin stiffness displayed the most potent modulation of cooperativity.

**Figure 4:**
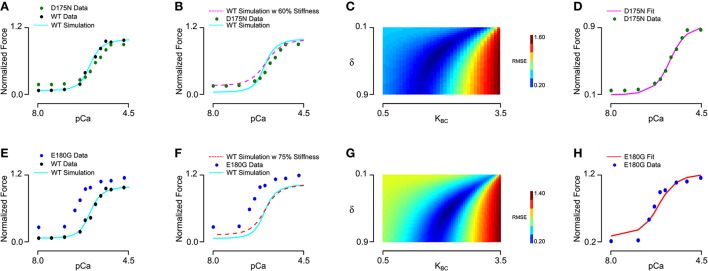
**Fitting of wild-type and mutant tropomyosin reconstituted myocardium steady state force production data. (A)** Baseline parameters were found that allowed the model to fit the wild-type data, with D175N tropomyosin mutation data shown for comparison. **(B)** In order to fit the D175N data, tropomyosin stiffness was first reduced by 40% and was not sufficient to fit the data. **(C)** Duty cycle and BC equilibrium were varied systematically while root mean square error of the model output and the D175N data was calculated, with an error minimum which maintained the baseline duty cycle but decreased the BC equilibrium constant by 15%. **(D)** The D175N simulation from a prescribed stiffness change and an optimized BC equilibrium decrease show a qualitative fit with the D175N mutant experiment data. **(E)** Wild-type data and the model fit shown, with E180G tropomyosin mutation data shown for comparison. **(F)** In order to fit the E180G data, tropomyosin stiffness was first reduced by 25% which was not sufficient to fit the data. **(G)** The error minimum for the E180G heatmap after applying the 25% stiffness reduction was found to include both a 10% increase in BC equilibrium and a 15% increase in duty cycle. **(H)** After applying the stiffness change, BC equilibrium change, and duty cycle change, the E180G simulation shows a qualitative fit with the experimental data for the E180G mutation.

We next sought to determine whether the effects of tropomyosin mutations on thin filament activation could be captured by altering tropomyosin-related model parameters. To do this, we analyzed a steady-state force-pCa dataset (Bai et al., 2011) measured in bovine cardiac muscle in which thin filament proteins were extracted and reconstituted with 100% human recombinant wild-type or mutant alpha tropomyosin. Importantly, the tension of the reconstituted filaments in that study was carefully measured with respect to a reference tension at 0°C in the relaxing solution, allowing analysis of not only the calcium dependent activation but also the calcium independent activation of the muscle. We began by fitting the wild-type data using a constrained particle swarm optimization and found a set of parameters (Table 2, Set 2) that produced a high fidelity simulation of the reconstituted fibers with wild-type tropomyosin (Figures 4A,E); the Hill coefficient and pCa_50_ of the wild-type simulation curve was almost identical to that of the experimental curve (Table 2). This parameter set was similar to the parameter set for skinned cardiac muscle (Set 1) but included a lower tropomyosin stiffness and non-zero lambda, which permits loose coupling of troponin and tropomyosin (Aboelkassem et al., 2015). These differences seem reasonable in light of the reconstitution process as well as species differences (rat vs. human/bovine). Previous electron microscopy studies and molecular dynamics simulations have established that E180G and D175N mutations increase the mean deviation angle of the tropomyosin coiled-coil from a wild-type value of 22.0 degrees to 27.6 degrees for E180G and 30.5 degrees for D175N (Li et al., 2012), representing a 25 and 40% decrease in stiffness respectively. We ran simulations by altering only the stiffness of the WT fit by these proportions (Figures 4B,F) and found that these changes alone were insufficient to reproduce the differences between the mutants and the wild-type data. This suggests that mutation effects are not confined to stiffness alone and that other molecular mechanisms should be considered. Indeed, tropomyosin mutants have previously been determined to alter the interactions between tropomyosin and the actin surface (Orzechowski et al., 2014a; Zheng et al., 2016), which may in turn affect both the blocked-to-closed and closed-to-open (myosin-induced) transitions of tropomyosin across actin. We therefore entertained the possibility that introducing changes to the BC equilibrium constant and duty cycle in addition to the assumed stiffness changes could produce a reasonable fit to mutant data. In order to be thorough, a two-dimensional parameter space for *K*_*BC*_ and δ was explored for each mutation (Figures 4C,D). In both cases, we found a global minimum in the error landscape, indicating constrained solutions for fitting the mutant force-pCa curves. For D175N, the optimal simulation demanded a decrease in the BC equilibrium by 15%, favoring the blocked state, in addition to the measured 40% decrease in tropomyosin stiffness (Figure 4D). For E180G, the optimal simulation required both an increase in the BC equilibrium by 10% and an increase in the myosin duty cycle by 15%, in addition to the reported 25% decrease in tropomyosin stiffness (Figure 4H). The model therefore suggests that the molecular consequences of a tropomyosin mutation can include changes in flexibility, the blocked-to-closed tropomyosin transition, and the crossbridge-mediated closed-to-open tropomyosin transition but varies relatively between mutations.

**Figure 5:**
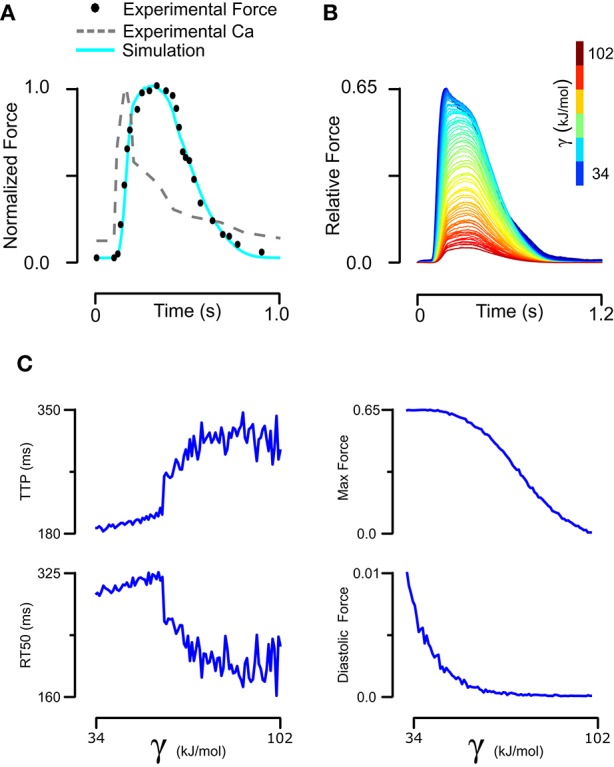
**Fitting of intact rat trabeculae twitch with its measured calcium transient and analysis of its sensitivity to stiffness. (A)** Baseline parameters maintaining the stiffness of skinned rat trabeculae were found that allowed a model simulation matching the experiment twitch while taking its real calcium transient as an input. **(B)** The baseline twitch was perturbed by variation of the tropomyosin stiffness. **(C)** Stiffness had complex effects on the force production and kinetics of twitch, with lower stiffness increasing maximum and diastolic force production while decreasing time to peak but increasing time from peak tension to 50% relaxation.

Understanding how mutation-driven changes in skinned fiber behavior might relate to the twitch characteristics of intact muscle is far from straightforward. The model provides a means of doing this, by extending the parameter changes determined in skinned fibers to predict intact muscle contraction. We first established a baseline set of intact muscle parameters (Table 1, Set 3) by fitting an isometric rat papillary muscle twitch record in response to its measured calcium transient (Janssen and de Tombe, 1997). The new model reproduced this dynamic calcium-activated response just as well as our previous (higher order) models (Figure 5A). In order to gauge the effects of tropomyosin stiffness on dynamic muscle activation, we ran twitch simulations with the same baseline parameter set and calcium transient while varying γ (Figure 5B). A striking result was the effect of stiffness on maximum activation of the muscle (Figure 5C). Increasing tropomyosin stiffness stunted force production potently above γ values of ~70 kJ/mol. At the same time, lowering stiffness resulted in higher fraction of the maximum force being present at diastolic calcium levels (Figure 5C). The kinetics of muscle activation as measured by time to peak force (TTP) and time from peak force to 50% relaxation of muscle (RT50) were also impacted (Figure 5C). Lowering stiffness tended to shorten TTP and increase RT50. Altogether, a more flexible tropomyosin resulted in increased maximum systolic force, modestly increased diastolic force, shortened TTP, and prolonged RT50.

**Figure 6:**
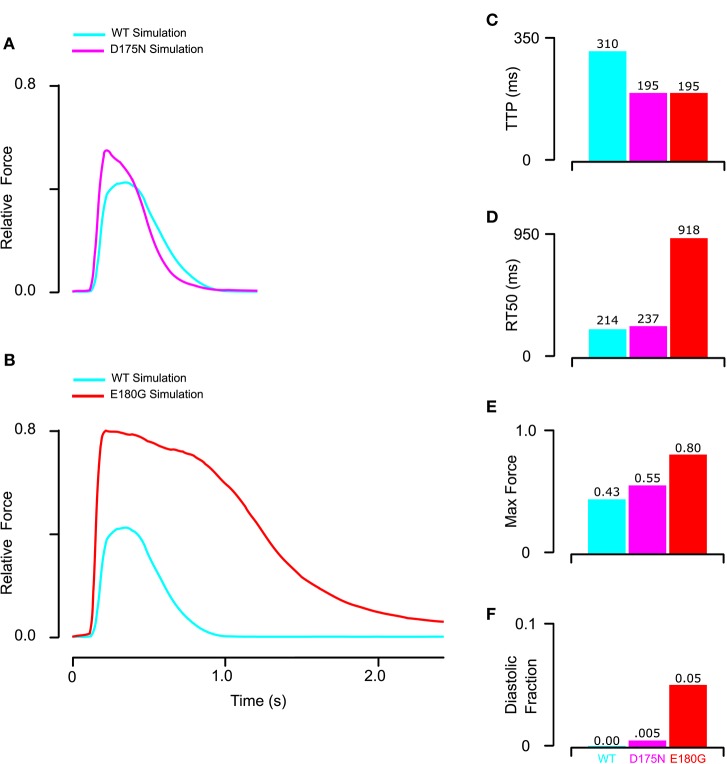
**Prediction of mutant twitch based on properties altered by mutant tropomyosin from fitting of the reconstituted myocardium data. (A)** D175N mutant twitch was predicted by applying a 40% increase in stiffness and a 15% decrease in BC equilibrium constant. **(B)** E180G mutant twitch was predicted by applying a 15% increase in duty cycle, 10% increase in BC equilibrium, and 25% reduction in stiffness. **(C)** Both E180G and D175N mutants reduced the time to peak to the minimum value possible in the model for the given measured calcium transient. **(D)** E180G and D175N increased the time from peak tension to 50% relaxation though D175N was predicted to be far more modest than E180G. **(E,F)** Both E180G and D175N mutants were predicted to increase maximum and diastolic force production though E180G had a larger effect.

Using model parameters (Table 1, Set 3) fitted to mutant tropomyosin fiber data (Figure 4), we predicted D175N and E180G effects on twitch force dynamics. For the mutant D175N, we applied a 40% decrease in stiffness and a 15% decrease in BC equilibrium constant (Table 1, Set 4) and found that the twitch was stronger overall with a small increase in diastolic force fraction (Figures 6A,E,F). Twitch kinetics were also altered, with a shorter TTP and longer RT50 (Figures 6C,D). For E180G, we applied a 25% increase in tropomyosin flexibility, 10% increase in BC equilibrium constant, and 15% increase in duty cycle (Table 1, Set 5). This yielded a twitch that had a much higher maximum force production and diastolic fraction. It also had a much shorter TTP and a much longer RT50 (Figures 6B–F). While the two twitches produced different properties, they both embodied faster activation, slower relaxation, and increased systolic and diastolic force compared to the wild-type.

## Discussion

We have considered the potential impacts of tropomyosin mutations on cardiac muscle function using a new structurally motivated thin filament model. Accounting for experimentally determined azimuthal shifts in tropomyosin (Poole et al., 2006) allowed us to reduce the dimensionality of our previous model (Aboelkassem et al., 2015) from 12 free parameters to just eight. In effect, four parameters that were considered independent are constrained by structural data and thermodynamic principles in the new formulation. Along with the reduced dimensionality, these constraints have made the model more realistic. It recapitulates observed features of cardiac muscle activation, including distinct Hill coefficients for upper and lower halves of the steady-state force-pCa curve (Figure 2B; Dobesh et al., 2002), strong dependence of the rate of force redevelopment on calcium (Figure 2A; Brenner, 1988; Fitzsimons et al., 2001) and realistic calcium-activated twitch transients (Figure 5A). As the model's parameters also directly relate to the molecular properties of tropomyosin, it provided a means for systematic investigation of HCM-linked tropomyosin mutations. Simulation-based analysis of skinned fiber data suggests that functional consequences of TPM1 mutations may be explained by three fundamental properties, namely tropomyosin chain stiffness, BC equilibrium, and tropomyosin-mediated changes in the crossbridge duty cycle.

Our results suggest that each tropomyosin mutation causes a unique combination of changes to these three molecular properties, and that specific changes can be estimated by model analysis when the right data are available. At first examination, the ability of skinned fiber exchange experiments to discriminate between tropomyosin molecular property changes seemed limited. For instance, the pCa value for half-activation of force (pCa_50_) is predicted to be highly sensitive to all three properties (Figure 3F). However, if the mutation effect on tropomyosin stiffness is known *a priori*, the model is able to predict its concomitant impact on pCa_50_, Hill coefficient, and maximum/minimum forces (Figures 4B,F). The remaining discrepancies with mutant data can then be reasonably ascribed to changes in actin-tropomyosin surface interactions, appearing as changes to the BC and CM equilibria. Although these two properties have similar effects on the steady-state force-pCa curve, only the CM equilibrium (i.e., crossbridge duty cycle) is predicted to significantly impact maximum calcium-activated tension. As a consequence, a simple two-parameter search was able to minimize error between the model and the mutant fiber data and yield unique parameter values for *K*_*BC*_ and δ (Figures 4C,G). Our approach was facilitated by recognizing that kinetic rate pairs in the model reduce to equilibrium coefficients when fitting steady-state measurements. The subsequent assumption that tropomyosin mutations do not directly affect the Ca^2+^ affinity of troponin C left only *K*_*BC*_ and δ as free parameters.

It is worth noting that the precision with which Bai et al. (2011) performed their skinned fiber studies with wild-type and mutant tropomyosins was critical to our approach. The calcium-free force produced in skinned preparations is often not reported due to technical challenges. Instead, force values at very low calcium concentrations are frequently assumed to be equivalent to zero. However, as our analysis demonstrates, force-pCa curves that are made to start at zero do not allow clear differentiation between competing biophysical changes such as tropomyosin stiffness and the apparent crossbridge duty cycle. Hence, while D175N and E180G mutations have been studied by others in various experimental systems (Bottinelli et al., 1998; Michele et al., 1999; Muthuchamy et al., 1999; Evans et al., 2000; Wang et al., 2011; Rysev et al., 2012), we selected the data of Bai et al. for the analysis here because it is the only applicable data set of which we are aware that reports absolute force measurements under very low calcium conditions.

Although tropomyosin stiffness can be directly studied via electron microscopy as well as atomic force microscopy (Li et al., 2010, 2012; Loong et al., 2012b) and simulated using molecular dynamics, no comparable approaches yet exist for obtaining estimates of the intrinsic BC and CM state equilibria. Hence, direct validation of our predictions for the E180G and D175N mutation effects on *K*_*BC*_ and δ are not possible at present. Instead, we must rely on indirect evidence to corroborate our estimates. For instance, in previous work, the electrostatic interactions was computed between tropomyosin and actin residues as tropomyosin is moved axially and azimuthally over the actin surface (Orzechowski et al., 2014a). This allows construction of energy landscapes for wild-type and mutant tropomyosins. In the absence of a reliable way to calculate entropic changes in this milieu, a direct estimate of the free energy changes between B and C states cannot be accurately made. However, qualitative insight is possible. We predicted from fitting fiber data that the E180G mutation would cause a 10% increase in *K*_*BC*_, indicating a relative increase in the free energy associated with the B state. This agrees well with the prediction of Orzechowski et al. (2014a) that the E180G substitution would cause a ~40 kJ/mol increase in the electrostatic potential of the B state. Corresponding analysis of the actin surface interactions of D175N tropomyosin showed the changes in that case were quite small. Although we found a global fit for D175N suggesting a 15% decrease in *K*_*BC*_ (Figure 4C), the slope of the error surface is sufficiently shallow that reasonable fits can also be obtained by assuming no change in *K*_*BC*_ and a slight decrease in δ. In light of the predicted absence of actin surface interaction changes and the shallowness of the error landscape (Figure 4C), we conclude that the majority of D175N effects are explained by reduced tropomyosin chain stiffness (Figure 4B). Placing less importance on equilibrium effects also seems prudent in light of at least one study (Evans et al., 2000) that reports increased myofilament Ca^2+^ sensitivity in mouse myocardium expressing D175N tropomyosin, as opposed to the unchanged pCa_50_ seen in the data of Bai et al. (2011). Increased Ca^2+^ sensitivity is consistent with the model-predicted effects of a pure decrease in tropomyosin stiffness (Figure 4B).

Another way of indirectly validating our predictions is to look at how E180G affects intact muscle twitches. Our model generally predicts outcomes that are consistent with those data. When predicting the effect of HCM-related mutations E180G and D175N on intact muscle, we found that E180G is more severe than D175N (starting from a rat twitch background and calcium transient). This aligns with observations that transgenic mice expressing E180G show a severe phenotype and often die within 6 months of age, while those expressing D175N display a milder phenotype (Redwood and Robinson, 2013). At the same time, both mutations were predicted in the model to cause increased diastolic and systolic tension. Similar behavior has been consistently demonstrated in studies of myocardium containing mutant tropomyosin. Isometric force measurements of permeabilized myocytes with both adenovirally expressed D175N and E180G showed high force production at low calcium (Michele et al., 1999). In terms of twitch kinetics, only E180G has been extensively characterized, but these results largely agree with our predictions. Papillary muscles isolated from transgenic E180G mice showed a slightly longer time to peak and a severe slowing of relaxation by almost 60% compared to control (Sheehan et al., 2011). Adult cardiomyocytes isolated from these animals showed increased magnitude of unloaded shortening of approximately 300% (Sheehan et al., 2011), which also agrees with our predictions.

One puzzling component to understanding pathogenicity of D175N tropomyosin is that is displays a reduced calcium sensitivity compared to wild-type, and no increase in maximally activated steady-state force (Bai et al., 2011). This seemingly contradicts the notion that HCM mutations are generally hypercontractile in nature. However, we find it interesting that our simulated D175N twitch is in fact hypercontractile (Figure 6A), with a predicted 28% increase in peak twitch force. According to the model, cooperativity-linked kinetic effects explain how D175N can increase twitch tension even while having a reduced steady-state calcium sensitivity. The large drop in tropomyosin stiffness associated with D175N means that nearest-neighbor RUs are less strongly coupled. At sub-maximal calcium levels (such as those occurring in a transiently-activated twitch event), weaker RU-RU coupling actually allows tension to develop more rapidly. Hence, although D175N ultimately limits the steady-state force, it develops force more quickly and thus reaches a higher level than wild-type during a short twitch event. This modeling result illustrates the limitations of using steady-state force-pCa curves and their properties as the sole means of understanding mutation pathogenicity.

In addition to the primary sarcomeric effects, HCM is characterized by myocardial remodeling, specifically concentric hypertrophy, fibrosis, and myocardial disarray (Teerkaririkul et al., 2012). Excess diastolic tension (Bai et al., 2013) as well as hypercontractility, may represent a common pathway for how tropomyosin mutations act to initiate HCM-type ventricular remodeling. Recent work suggests that increased myofilament tension during the twitch cycle, calculated as the scalar tension-time integral, induces ERK1/2 signaling leading to HCM-like hypertrophic remodeling (Davis et al., 2016). Our twitch predictions for E180G and D175N both achieve higher peak tension, and have tension-time integrals that exceed wild-type. Even though the final mutant twitches appear quite different from each other, the tension-time integral could explain why both are capable of driving concentric cardiomyocyte hypertrophy. Reducing myofilament activation in E180G mice by either increasing calcium uptake through knocking out phospholamban (Gaffin et al., 2011) or by co-expression of pseudophosphorylated TnI (Alves et al., 2014) was previously shown to decrease ERK1/2 signaling and prevent pathological hypertrophy. Furthermore, our model suggests than D175N would appear milder than E180G when it comes to these metrics, which is consistent with evidence that E180G has much more severe remodeling and disease than D175N (Redwood and Robinson, 2013).

While the twitch predictions show fairly extreme changes in the presence of mutations, particularly for E180G, it should be noted that these are the simulated effects of 100% replacement of wild-type tropomyosin with mutant tropomyosin. The clinical scenario is certainly different. In studies of HCM patients, monoallelic D175N is expressed to a similar amount as wild-type (Bottinelli et al., 1998) which would lead to coiled-coils consisting of a presumed mixture of 25% wild-type homodimers, 25% mutant homodimers, and 50% heterodimers. Recent biochemical studies with E180G and D175N show that the 1:2:1 ratio of dimers is formed in solution and that there was only a very slight preference for wild-type versus mutant tropomyosin when it came to binding of actin (Janco et al., 2012). However, for other properties of heterodimers such as calcium sensitivity, there was not necessarily a consistent trend of heterodimers mimicking homodimers (Janco et al., 2012). Therefore, our model utilizing complete replacement of wild-type homodimers with mutant homodimers would predict the worst case scenario. Furthermore, our model does not take into account any cellular adaptation that may occur as a result of a sarcomeric perturbation, such as tropomyosin phosphorylation, calcium transient changes through regulation such as phospholamban, and other myofilament post-translational changes (Teerkaririkul et al., 2012; Redwood and Robinson, 2013). Presumably, some of these adaptations may act to alleviate the functional phenotype and lead to milder dysfunction than predicted.

Although the model provides valuable insights, it is important to consider some of its limitations. Like our previous models, we employed an RU-level discretization of the thin filament. This means that any effects arising from intra-RU phenomena are absent and that effectively only one myosin crossbridge is represented per RU (see discussion in Campbell et al., 2010). Also, at present the model is only capable of simulating isometric contraction. We did not explicitly represent tropomyosin-troponin T interactions in the model, even though recent evidence has emerged suggesting their potential modulation by tropomyosin mutations (Orzechowski et al., 2015) and possible effects on tropomyosin chain stiffness (Sousa et al., 2010). Such interactions are still of unclear significance with regards to thin filament regulation, but could explain some shortcomings of the present model. Specifically, the model deviates from the measured E180G exchange data at low Ca^2+^ concentrations (Figure 4H). The only way of reconciling this deviation is to assume that the drop in overall tropomyosin chain stiffness is not as large as that measured for individual E180G tropomyosin dimers. It is possible that in the presence of troponin T the effect of E180G on stiffness is reduced. With the appropriate structural information, tropomyosin-troponin T interactions could be included in future modeling efforts.

Of possible consequence to the present study, the discretized thin filament requires us to translate proportional tropomyosin stiffness into energy via a simple unidimensional deformation. As such, we do not account for some of the more complex motions of tropomyosin geometry as considered in continuous flexible chain type models (Smith and Geeves, 2003; Smith et al., 2003; Mijailovich et al., 2012; Metalnikova and Tsaturyan, 2013; Land and Niederer, 2015). One possible consequence of leaving out this complexity is that the simplified model might not behave in a realistic matter. Given the model's ability to produce several fundamental muscle phenomena, any such limitations seem likely to be second order in nature. Another possibility is that the simplified model yields realistic behavior but can only do so when assuming unrealistic parameter values. To examine this, we compared our estimate of tropomyosin chain bending stiffness per unit length (obtained by fitting cardiac muscle force-pCa data) to the estimate used by Smith et al. (2003) and Smith and Geeves (2003). They estimated a value of 2.5 × 10^−44^ Nm^4^ for the bending stiffness per unit length; while using our fitted value for the apparent chain stiffness (Table 1), we would back calculate a value of 1.3 × 10^−44^ Nm^4^. Considering the simplifications involved, the agreement of these values to well within an order of magnitude seems reasonable.

A few additional details surrounding the exchange experiments of Bai et al. (2011) should be considered. They used recombinant human tropomyosin with an added Gly-Ser sequence at the N-terminus to approximate acetylation (Monteiro et al., 1994). N-terminal acetylation of tropomyosin stabilizes its coiled-coil structure and hence could be an important determinant of overall tropomyosin chain stiffness (Greenfield et al., 1994). Although the work of Monteiro et al. (1994) that the dipeptide extension is functionally equivalent to acetylation, there may still be differences between native and recombinant tropomyosins. This may explain why values for the effective tropomyosin chain stiffness (γ) fit to skinned rat cardiac tissue data differ from those fit to bovine myocardium reconstituted with recombinant human tropomyosin. Another factor to contemplate is that the various experimental data sets were acquired at different temperatures. In order to ascribe the effects of tropomyosin mutations on γ and other parameters as uncovered by fitting to the data of Bai et al. (2011) we computed their proportional effect and then applied the same proportional changes to the baseline parameters fitted to intact rat cardiac twitch data.

Although significant progress has been made already toward modeling the thin filament and tropomyosin at different scales, we present here the first attempt of which we are aware to model tropomyosin mutations in an integrative manner and predict their effects on intact muscle function. In the future, the spatially discretized thin filament approach will also allow simulations with stochastic assembly of wild-type and mutant homodimers and heterodimers which may have important implications for pathogenesis (Janco et al., 2012). We are also continuing work toward a discretized coarse-grain model of tropomyosin and incorporation of sarcomere length dependence, which will improve the ability of the model to translate biophysical alterations of tropomyosin into functional simulations.

## Author contributions

LS: Derived the model, implemented the model, designed and ran simulations, and wrote the paper; JM: Assisted with model derivation and writing of the paper; WL: Assisted with model derivation and writing of the paper; SC: Derived the model, designed simulations, and wrote the paper.

### Conflict of interest statement

The authors declare that the research was conducted in the absence of any commercial or financial relationships that could be construed as a potential conflict of interest.
